# Results from a clinical yoga program for veterans: yoga via telehealth provides comparable satisfaction and health improvements to in-person yoga

**DOI:** 10.1186/s12906-017-1705-4

**Published:** 2017-04-04

**Authors:** R. Jay Schulz-Heik, Hilary Meyer, Louise Mahoney, Michael V. Stanton, Rachael H. Cho, Danae P. Moore-Downing, Timothy J. Avery, Laura C. Lazzeroni, Joanne M. Varni, Linda Martin Collery, Peter J. Bayley

**Affiliations:** 1 War Related Illness and Injury Study Center, VA Palo Alto Healthcare System, 3801 Miranda Avenue, Palo Alto, CA 94301 USA; 2 Palo Alto University, 1791 Arastradero Road, Palo Alto, CA 94304 USA; 3 Department of Psychiatry and Behavioral Sciences, Stanford University, 450 Serra Mall, Stanford, CA 94305-2004 USA

**Keywords:** Yoga, Telehealth, Feasibility, Effectiveness

## Abstract

**Background:**

Yoga is increasingly popular, though little data regarding its implementation in healthcare settings is available. Similarly, telehealth is being utilized more frequently to increase access to healthcare; however we know of no research on the acceptability or effectiveness of yoga delivered through telehealth. Therefore, we evaluated the feasibility, acceptability, and patient-reported effectiveness of a clinical yoga program at a Veterans Affairs Medical Center and assessed whether these outcomes differed between those participating in-person and those participating via telehealth.

**Methods:**

Veterans who attended a yoga class at the VA Palo Alto Health Care System were invited to complete an anonymous program evaluation survey.

**Results:**

64 Veterans completed the survey. Participants reported high satisfaction with the classes and the instructors. More than 80% of participants who endorsed a problem with pain, energy level, depression, or anxiety reported improvement in these symptoms. Those who participated via telehealth did not differ from those who participated in-person in any measure of satisfaction, overall improvement (*p* = .40), or improvement in any of 16 specific health problems.

**Conclusions:**

Delivering yoga to a wide range of patients within a healthcare setting appears to be feasible and acceptable, both when delivered in-person and via telehealth. Patients in this clinical yoga program reported high levels of satisfaction and improvement in multiple problem areas. This preliminary evidence for the effectiveness of a clinical yoga program complements prior evidence for the efficacy of yoga and supports the use of yoga in healthcare settings.

## Background

Over the past two decades, yoga has gained in popularity as an adjunct mind-body treatment for a range of mental and physical health issues [1]. While a growing number of randomized controlled trials of yoga have been published [2–4]. few studies have evaluated the feasibility, acceptability and effectiveness of yoga programming in real-world clinical settings. In parallel to the increasing popularity of yoga, new information technologies such as telehealth are revolutionizing healthcare. This article evaluates a clinical yoga program which has been offered to Veterans at the VA Palo Alto Health Care System for several years both in-person and via telehealth. It describes patients’ satisfaction with and perceived health improvements from their participation in the program. It also compares outcomes from a telehealth version of the program, through which a yoga class is taught to patients at remote locations via videoconference, to that from an in-person version of the program and it discusses support for the feasibility of yoga for treating diverse health issues in Veterans.

A rigorous review concluded that there is generally insufficient research to support the use of yoga as a stand-alone intervention for many conditions, though there is more significant empirical support for its treatment of chronic low back pain [5, 6]. There are several reasons yoga may be particularly suitable as an adjunct therapy for Veterans. First, Veterans often present with complex physical, psychological, and social health problems. For example, a recent study of over 200,000 Veterans returning from Iraq and Afghanistan found that 36.9% were diagnosed with a mental health disorder, including posttraumatic stress disorder (PTSD; 21.8%) and depression (17.2%) [7]. The rates of these disorders are increasing [8] and are associated with high rates of physical illness such as cardiovascular disease and cancer [8–11] as well as interpersonal difficulties, substance abuse, homelessness, and suicide [8].

Yoga may be suitable for Veterans with multiple comorbid conditions as similar yoga programs have been associated with diverse positive impacts [12]. Further yoga involves both physical and psychological mechanisms of action. For example, converging evidence suggests that yoga can help regulate the hypothalamic-pituitary-adrenal axis and balance neuroendocrine, immune, and metabolic systems [13, 14]. It is also thought to promote psychological processes of concentration and mindfulness [13, 14], decentering [15], and emotion regulation [16]. Therefore it may improve a vast array of physical and mental health problems.

Second, with large numbers of Veterans returning from recent conflicts, there is an increased need for cost-effective interventions that can treat this growing population [9]. For example, 40% of Veterans from recent conflicts have utilized VA care, while only 10% of Vietnam Veterans sought VA care [8]. Given the high costs and limited effectiveness of practitioner-delivered interventions, self-care techniques are being advocated as a more sustainable alternative to explore [17]. This is consistent with a “patient centered” approach in which care is tailored to individual characteristics and conditions. In this model, patients are offered complementary and integrative medicine (CIM; e.g. yoga, tai chi, meditation) to supplement or replace standard medical treatment. Indeed, one of the reasons for the increase in the provision of mind-body practices such as yoga within the VA system is that the VA recognizes complementary approaches to healthcare can empower Veterans to improve their own well-being [18]. Yoga may be cost-effective [2] for two reasons. First, it can be provided to a group, and thus as a one-to-many rather than one-to-one intervention. Second, it may function as a preventive intervention that stops or slows the development of costly chronic illnesses [19].

Third, new information technologies are revolutionizing healthcare and offer large potential benefits to Veterans and other patients. Telehealth is one such technology with potential benefits that include improved access to healthcare, especially those with limited mobility or those living in rural areas, decreased travel time to a clinic, and the containment of treatment costs. The Office of Telehealth Services in the VA has focused on four applications of telehealth including Telemental Health, Telerehabilitation, Teleretinal screening, and Primary Care Telehealth Outreach Clinics. Across these programs, telehealth has been growing steadily since 2005, and over 140,000 unique patients were projected to receive services via telehealth in 2012 [20]. Although the delivery of yoga via telehealth has not been widely evaluated, several published studies of the treatment of mental health issues such as PTSD via telehealth indicate that it is broadly accepted, effective, and more cost-effective than in-person interventions [21–27].

The War Related Illness and Injury Study Center (WRIISC) program at the VA Palo Health Care System has provided a yoga program to Veterans at its Palo Alto location since 2010. A Clinical Video Telehealth (“Telehealth”) component was added beginning in 2012. Referrals to both programs have increased each year bringing the cumulative number of referrals to more than 2300 as of 2016. Telehealth involves the use of a secure real-time interactive video conferencing system that links the patient(s) at an outlying clinic to a provider at another location. In the WRIISC yoga program, an instructor located in the Palo Alto Division provides yoga instruction to students who are in the same room. These classes are often simultaneously broadcast via the videoconferencing system to students at outlying community-based outpatient clinics. Many students report enjoying and benefitting from the classes. However, little empirical evidence for the effectiveness of the program exists. Therefore, in order to assess the feasibility, acceptability, and self-reported effectiveness of the program here we report students’ satisfaction with the program and their self-rated improvement in physical and mental health. We also compared these outcomes between those who attended in-person and those who attended via telehealth.

## Method

### Participants and procedure

The yoga program currently offers 13 classes per week - eight classes are open drop-in outpatient classes (including three classes that are simultaneously provided as telehealth yoga) and five additional classes that are restricted to patients in a specific setting (e.g. in an inpatient psychiatry unit or residential trauma treatment program and not simultaneously offered via telehealth). Attendees report being motivated to attend by a wide variety of health conditions and/or to enhance well-being. Outpatient students are referred by VA staff and receive clearance from a medical provider and, when applicable, a mental health care provider. Students in residential or inpatient programs are cleared to participate in yoga classes by the program’s medical providers. Some students request a referral from their provider while others attend on the recommendation of their providers. Classes are taught by five different Yoga Alliance-certified instructors with a minimum of 200 h of teaching experience who have training in different lineages of hatha yoga, in addition to in-house training on teaching yoga to the Veteran population. Most of the yoga instructors have additional training and teaching experience. All instructors utilize a standard WRIISC protocol which incorporates meditation, intention setting, controlled breath practices, postures emphasizing alignment, synchronization of breath and movement, and relaxation/meditation. Classes are one-hour long and utilize equipment such as mats, blocks, straps, blankets and chairs to adapt postures to the needs of each student.

All classes are taught at the Palo Alto Division. During the classes enhanced with the videoconference connection to the outlying clinics, the instructor can see the students at the outlying clinics on a screen and provide appropriate feedback. Students at the outlying clinics can see the instructor on their screen. For safety reasons, classes offered as simultaneous in-person and telehealth classes are limited to chair-based yoga. In these classes the students sit on chairs for the meditation, warm-up, and final relaxation portion of the class and have the option to sit or stand for the more active portion of class.

Students are free to attend as many of the open outpatient classes as they wish and some students attend between 2 and 4 classes per week. The telehealth classes are provided to patients at six remote clinics within the VA Palo Alto Health Care System and are available up to 3 days/week (2 days per week for all genders, 1 day per week for women only).

To assess students’ satisfaction and symptom improvement, all participants who attended any class within a two week period in March 2015 were invited to complete a self-report survey. Students are not limited to a fixed number of sessions. Therefore, this evaluation includes data from participants who have been attending WRIISC yoga classes for months or years as well as participants for whom this is a first yoga class and all cases in-between. In order to facilitate candid reporting, identifying information such as respondents’ names was not collected.

The anonymous survey data was collected for program management and evaluation purposes. Therefore, consistent with policy at the university which provides ethics review for the program site, no ethics review of the survey was conducted.

### Measure

The survey included multiple choice items assessing Veterans’ demographics, their satisfaction with the classes they attended, and the extent to which they noticed improvements in various aspects of their health. To preserve anonymity, the only demographics that were collected were gender and the era in which they served in the military. A survey item assessed the number of WRIISC yoga classes the respondent has taken. Answer options were defined in order to capture the range of student experience. Items assessing satisfaction included ‘Overall, how would you rate the quality of your yoga class?”, ‘How would you rate the quality of the yoga classroom and equipment?’ and ‘Did your yoga teacher explain things in a way that was easy to understand?’ These items were rated on a four point scale ranging from ‘poor’ to ‘excellent’ or ‘not at all’ to ‘yes, completely.’ Items assessing improvement in symptoms included one item assessing global health improvement and 16 items assessing improvement in specific symptoms, such as back pain, trouble falling asleep, and anxiety. The five answer options for these 16 items were; “not applicable”, ‘no improvement’, ‘not very much’, ‘yes, somewhat’ and ‘yes, completely.’

### Analyses

#### Overall program feasibility, acceptability and effectiveness

Frequencies of demographic and satisfaction variables were tabulated. The 16 symptom-improvement variables were dichotomized: ‘no improvement’ and ‘not very much’ .were counted as not improved and ‘yes, somewhat’ and ‘yes, completely’ were counted as improved. The proportions of Veterans who reported improvement in each symptom were computed. Veterans who reported not having a particular problem were not included in the summary statistics for that symptom. Whether the percent improved was statistically significantly greater than a threshold was tested. Since the intervention is low cost, it may be an appropriate activity even if only a moderate proportion of Veterans improve through their participation. Therefore a low comparison threshold of >33% improvement was selected and tested using a nonparametric one-sample binomial test.

#### In-person vs. Telehealth

To compare satisfaction and improvement between in-person and telehealth modalities, independent samples nonparametric tests or chi-square analyses were conducted, as appropriate. Following convention, a primary alpha of .05 was chosen. Given the 16 problem areas assessed for improvement, a Bonferroni correction was used to set a secondary alpha = .003 (i.e., .05 /16).

## Results

### Full sample demographics, satisfaction and improvement

In 2015, the year in which the survey was administered, 719 Veterans attended at least one WRIISC yoga class, which represented about 1.4% of all Veterans served by the VA Palo Alto Health Care System. Table 1 shows respondents’ demographic and yoga participation data. Similar numbers of Veterans attended classes in-person (*n* = 29) and via telehealth (*n* = 30). Of the 64 Veterans who completed the survey, almost three quarters were male (*n* = 47). About half of the Veterans served in the military during the Vietnam war era (*n* = 29). About one-fifth served during the recent conflicts in Iraq and Afghanistan (Operation Iraqi Freedom and Operation Enduring Freedom; *n* = 11). The number of WRIISC yoga classes Veterans attended ranged widely, with 40% of the sample having attended five or fewer classes, and 29% having attended 21 or more classes. Gender, era of military service and number of yoga classes completed did not differ statistically significantly between groups. However, a trend was observed, with Vietnam era Veterans slightly more likely to attend via telehealth and Iraq and Afghanistan war Veterans slightly more likely to attend in-person.

**Table 1:** Demographics and yoga participation

	Total		In-Person		Telehealth		In-Person vs. Telehealth^1^
	*n*	%	*n*	%	*n*	%	*p*
*N*	64	100%	29	48%	30	50%	
*Gender*							
Male	47	73%	23	79%	20	67%	.28
Female	17	27%	6	21%	10	33%	
*Era*							
Vietnam	29	48%	10	35%	16	53%	.06
Desert Storm	11	18%	4	14%	6	20%	
Iraq or Afghanistan War	11	18%	8	28%	2	7%	
Multiple eras or other era	9	14%	7	24%	2	7%	
Korean War	1	2%	0	0%	1	3%	
*# of yoga classes attended*							
1–5	21	40%	12	55%	6	24%	.24
6–10	10	19%	2	9%	8	32%	
11–15	5	10%	1	5%	3	12%	
16–20	1	2%	0	0%	1	4%	
21+	15	29%	7	32%	7	28%	

^1^Chi-squared analyses for categorical data or Mann-Whitney U test for ordinal data, as appropriate

Table 2 displays respondents’ satisfaction with the yoga classes they attended. Respondents generally reported being very satisfied with the quality of the yoga classes, with 82% rating it ‘excellent.’ Almost all (98%) would recommend yoga classes to a friend. Almost all respondents (96%) reported feeling at least somewhat better after class than before, with the benefit most frequently lasting for a duration of between several hours and the remainder of the day. Almost all respondents (98%) endorsed enjoying the class. Similarly, almost all (98%) reported ‘completely’ feeling like they were treated with respect and dignity by the teacher. Relatedly, respondents reported that the teachers’ explanations were easy to understand. Interestingly, ratings of the quality of the classroom and equipment were somewhat lower, with about one-third rating it as ‘good’ or worse.

**Table 2:** Satisfaction

	*n*	%
Overall, how would you rate the quality of your yoga classes?		
excellent	51	82%
very good	11	18%
good/fair	0	0%
poor	0	0%
Would you recommend WRIISC yoga classes to a friend?		
yes	55	98%
no	1	2%
Do you feel better after your yoga class than you did before class?		
yes very much	39	63%
yes somewhat	21	34%
not very much	1	2%
not at all	1	2%
If so, how long does it last?		
the rest of the week	5	10%
the rest of the day	21	40%
several hours	17	33%
just an hour or two	9	17%
Do you enjoy the WRIISC yoga class?		
yes	61	98%
no	1	2%
Do you feel like you are treated with respect and dignity by the yoga teacher?		
completely	62	98%
mostly	1	2%
not completely	0	0%
not at all	0	0%
Did your yoga teacher explain things in a way that was easy to understand?		
completely	57	89%
mostly	7	11%
not completely	0	0%
not at all	0	0%
How would you rate the quality of the yoga classroom and equipment?		
excellent	28	46%
very good	14	23%
good/fair	17	28%
poor	2	3%

**Fig. 1:**
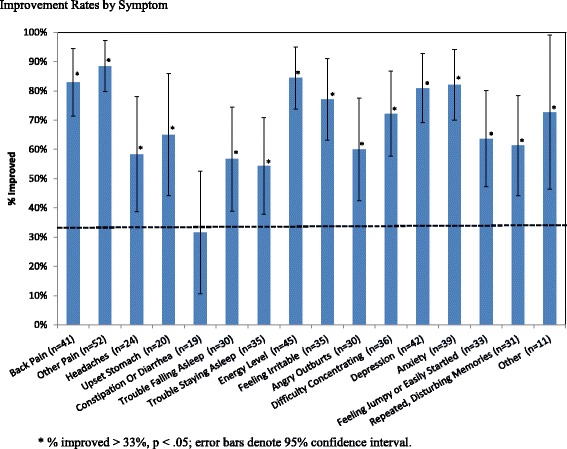
Improvement Rates by Symptom

In response to the item assessing overall improvement in symptoms, 12 respondents (26%) indicated complete improvement, 29 (62%) reported partial improvement and only 6 (13%) reported no improvement. Figure 1 displays rates of subjective improvement for each of the 16 specific problem areas. The most frequently endorsed problem areas - back pain, other pain, energy level, depression and anxiety – were all reported as improved at a rate of 80% or higher. Table 3 includes reported improvement rates, which ranged from 32% for constipation or diarrhea to 88% for pain other than back pain with a median of 69%. Subjective improvement rates were statistically significantly greater than 33% for all problems except for constipation or diarrhea at the *p* < .05 level and for 11 out of the 16 problem areas at the Bonferroni-corrected *p* < .003 level.

**Table 3:** Improvement

	Total			In-Person		Telehealth		In-Person vs. Telehealth		
	Improved/Total	% (95% CI)	*p* ≯ 33%	Improved/Total	% (95% CI)	Improved/Total	% (95% CI)	∆%	χ^2^	*p*
Back pain	34/41	83 (71–95)	<.001**	14/16	88 (69–100)	19/23	83 (66–99)	−.05	0.17	1.00
Other pain	46/52	88 (79–97)	<.001**	18/22	82 (64–99)	25/27	93 (82–100)	.11	1.31	.39
Headaches	14/24	58 (37–80)	.01*	9/11	82 (54–100)	5/11	45 (10–81)	−.36	3.14	.18
Upset stomach	13/20	65 (42–88)	.00*	5/8	63 (19–100)	7/11	64 (30–98)	.01	0.00	1.00
Constipation or diarrhea	6/19	32 (09–55)	.54	5/10	5 (12–87)	1/8	13 (0–42)	−.38	2.81	.09
Trouble falling asleep	17/30	57 (38–75)	.007*	8/15	53 (24–81)	7/12	58 (26–91)	.05	0.07	1.00
Trouble staying asleep	19/35	54 (37–72)	.008*	7/16	44 (16–71)	10/16	63 (36–89)	.19	1.13	.29
Energy level	38/45	84 (73–95)	<.001**	17/20	85 (68–100)	18/22	82 (64–99)	−.03	0.08	1.00
Feeling irritable	27/35	77 (63–92)	<.001**	11/16	69 (43–94)	13/16	81 (60–100)	.13	0.67	.69
Angry outburts	18/30	60 (41–79)	.002**	9/16	56 (28–83)	8/12	67 (35–98)	.10	0.31	.71
Difficulty concentrating	26/36	72 (57–88)	<.001**	13/18	72 (49–95)	11/15	73 (48–99)	.01	0.01	1.00
Depression	34/42	81 (69–93)	<.001**	14/19	74 (51–95)	18/20	90 (76–100)	.16	1.76	.24
Anxiety	32/39	82 (69–95)	<.001**	16/20	80 (60–99)	14/16	88 (69–100)	.08	0.36	.67
Feeling jumpy or easily startled	21/33	64 (46–81)	<.001**	10/16	63 (35–89)	9/14	64 (36–93)	.02	0.01	1.00
Repeated, disturbing memories	19/31	61 (43–79)	.001**	10/16	63 (35–89)	7/12	58 (26–91)	−.04	0.05	1.00
Other	8/11	73 (41–10)	<.001**	5/8	63 (19–100)	0/1	-	−.63	1.07	1.00

* *p* < .05; ** *p* < .003

### In-person vs. Telehealth

Table 4 displays satisfaction with yoga data for the in-person and telehealth groups. There were no statistically significant differences between groups. Most variables were rated uniformly highly across groups, and the smaller number of respondents in the telehealth group limited statistical power to detect differences.

**Table 4:** Satisfaction In-person Versus Telehealth

	In-person		Telehealth		
	*n*	%	*n*	%	*p* ^1^
Overall, how would you rate the quality of your yoga classes?					
excellent	25	89%	11	92%	.82
very good	3	11%	1	8%	
good/fair	0	0%	0	0%	
poor	0	0%	0	0%	
Would you recommend WRIISC yoga classes to a friend?					
yes	24	96%	28	100	.47
no	1	4%	0	0%	
Do you feel better after your yoga class than you did before class?					
yes very much	18	67%	9	75%	.68
yes somewhat	8	30%	2	17%	
not very much	0	0%	1	8%	
not at all	1	4%	0	0%	
If so, how long does it last?					
the rest of the week	1	3%	1	3%	.82
the rest of the day	12	41%	5	17%	
several hours	8	28%	4	13%	
just an hour or two	3	10%	1	3%	
Do you enjoy the WRIISC yoga class?					
yes	28	97%	28	100%	1.00
no	1	3%	0	0%	
Do you feel like you are treated with respect and dignity by the yoga teacher?					
completely	29	100%	11	100%	1.00
mostly	0	0%	0	0%	
not completely	0	0%	0	0%	
not at all	0	0%	0	0%	
Did your yoga teacher explain things in a way that was easy to understand?					
completely	27	93%	12	100%	.36
mostly	2	7%	0	0%	
not completely	0	0%	0	0%	
not at all	0	0%	0	0%	
How would you rate the quality of the yoga classroom and equipment?					
excellent	16	55%	4	33%	.32
very good	4	14%	3	25%	
good/fair	8	28%	5	42%	

^1^This column presents *p*-values for chi-square tests for categorical variables and Mann-Whitney U tests for ordinal variables

Veterans’ responses to the item assessing their overall symptom improvement since starting yoga classes did not differ between groups. Eighty eight percent of those participating in-person reported at least some improvement overall, as compared to 89% of those participating via telehealth (*p* = .97). Table 3 includes subjective symptom changes for both groups. For the 16 problem areas, the differences in % improved between groups were generally small and no problem areas were statistically significantly different. A greater portion of the in-person group improved in headaches (82%) and constipation or diarrhea (50%) than the telehealth group (45% and 13%, respectively), though these were not statistically significantly different likely due to particularly small sample sizes (*n* = 22, *p* = .18 and *n* = 18, *p* = .09, respectively).

## Discussion

Few studies have focused on the feasibility, acceptability and effectiveness of yoga programs within real-world hospital or community clinic settings. Overall, students in a clinical yoga program at our VA Medical Center were highly satisfied with the program, and 88% of respondents reported some degree of subjective symptom improvement. While yoga is available at many VA sites [28] there is a great deal of regional variability and the overall provision of yoga is sparse. Moreover, the majority of published yoga studies have focused on specific health problems such as back pain or depression [5] rather than the broad health benefits of yoga programs [29]. The present evaluation did not restrict respondents to those with any particular diagnosis and thus it provides preliminary support for the effectiveness of incorporating yoga into clinical programming for Veterans with a range of clinical presentations. Interestingly, pain (“in muscles, joints, or bones” and “back pain”), energy level, depression, and anxiety were the most frequently endorsed categories and each of these symptoms improved in at least 80% of patients by patient report. This observation suggests that yoga led to improvements in physical as well as psychological symptoms, and is consistent with the idea that yoga promotes both physical and psychological wellbeing [2] and may be appropriate for populations with high rates of physical and mental health co-morbidities.

Participants’ reports of high levels of satisfaction support the conclusion that the yoga program was feasible and well-accepted by patients, whether delivered in-person or via telehealth. Feasibility is further demonstrated by the increasing demand for classes and the ensuing robust schedule of 13 classes per week as well as the rarity of adverse events. According to the commonly accepted definition of serious adverse events [30], no students experienced any serious adverse events during the six year history of the program. While the majority of survey respondents were male, relative to the general population of VA patients, females were overrepresented in our sample. This may be due to classes being offered in women’s programs or to broader cultural factors [5, 31] and raises the possibility of a difference in the acceptability of yoga between genders among Veterans. There was a nonsignificant trend for Vietnam era Veterans to attend via telehealth yoga rather than in-person yoga. This may reflect Vietnam era Veterans being older and perhaps less mobile than younger Veterans. If confirmed in a larger controlled study, this observation would demonstrate the benefit of telehealth in reaching populations that otherwise may not participate in treatment. This observation awaits further study.

Prior randomized controlled trials have demonstrated yoga to be efficacious in treating a variety of medical and mental health conditions [2, 3, 32]. The present findings complement this research by providing preliminary support for the effectiveness of a yoga program in a healthcare setting. Our results are noteworthy in that a diagnostically diverse group of patients were highly satisfied and reported improvement in diverse symptoms. While providers should adapt and match interventions to different clinical disorders, the results suggest that yoga may be acceptable and feasible for a variety of chronic conditions, including pain, fatigue, and psychological distress (see also [12, 33]). Additionally, yoga’s ability to benefit a range of problems may enable health systems to efficiently provide services to patients with diverse presentations via a single intervention.

There is minimal published research on telehealth yoga, and to our knowledge this is the first report on this modality in Veterans. One recent study evaluated the feasibility of an eight-week Tele-Yoga program for participants recovering from heart failure and chronic obstructive pulmonary disease [34]. The study showed that while the program was appropriate and well-tolerated, technological problems and poor video quality caused difficulty. In the present program evaluation there were no differences in satisfaction or self-reported symptom improvement between the in-person and telehealth modalities. This observation encourages the continued expansion of telehealth to reach patients, particularly those who have been traditionally out of reach for geographic, clinical, financial, or other reasons. One idea that could be further explored is that telehealth may be particularly helpful in reaching a younger cohort of patients who may have limited time or be more comfortable seeking and utilizing care through technology.

Nevertheless, limitations of telehealth modalities should be considered. First, technological problems such as poor video quality or streaming can interfere with the ability to provide care [34]. This could be particularly problematic if they occur in situations with clinical risk in which patients may require immediate care. It should be noted, however, that in our program Veterans receiving telehealth were at outpatient community centers, not at home. Should clinical emergencies have arisen, clinicians were onsite and available to assist. Via this method or others, contingencies for risk need to be built into yoga programs. Second, delivering yoga via telehealth requires adequate space for all students, chairs and other equipment to fit in the camera’s view. Third, when providing yoga through telehealth, the fact that the instructor is not physically present with participants precludes him or her from making adjustments to students’ form in a manner involving physical touch. Rather, instructors are limited to making individual- and group-level adjustments via other means, such as verbal instruction or demonstration.

Several limitations require that these program evaluation results be viewed as preliminary. No control group was used, and as such it cannot be concluded that the improvements seen were due to the yoga program. Another limitation is that patients were not randomly assigned to in-person or telehealth conditions. A research study in which participants were randomly assigned to modality could more precisely assess for differences between modalities. The evaluations were subjective ratings rather than validated clinical measures. In this regard, it should be noted that participants in other yoga studies have reported subjective positive experiences and perceived changes even when measured changes were not significant [35]. The present evaluation would be complemented both by qualitative research exploring Veterans’ understanding of how yoga may be helpful and by quantitative research evaluating the acceptability and effectiveness of yoga via standardized assessments. Also, Veterans who attended classes during the week in which data was collected may not have been representative of all patients in the program. Future efforts may seek to reduce sampling bias, perhaps by sampling consecutive referrals rather than attendees. Finally, the fact that all telehealth classes were chair-based may have served as a potential confound when comparing the in-person versus telehealth programs. A future rigorous comparison of in-person versus telehealth classes should provide class conditions that are identical.

In sum, respondents’ ratings of satisfaction and symptom improvement provide preliminary evidence for the acceptability, feasibility and effectiveness of incorporating yoga into clinical programming for Veterans with a wide variety of physical and mental health problems. These findings provide preliminary support for the continued development and evaluation of yoga programs within healthcare systems. Ongoing clinical yoga programs should conduct feasibility and effectiveness studies to inform future programming’s development, dissemination and evaluation. Delivering yoga via telehealth appears to be acceptable and feasible. While doing so, issues such as risk management and treatment individualization should be carefully considered.

## Abbreviations

PTSD: Posttraumatic stress disorder
WRIISC: War Related Illness and Injury Study Center
